# Impact of Leaf Development Stages on Polyphenolics Profile and Antioxidant Activity in* Clausena lansium* (Lour.) Skeels

**DOI:** 10.1155/2018/7093691

**Published:** 2018-06-06

**Authors:** Xiaoxiao Chang, Yusheng Lu, Zhixiong Lin, Jishui Qiu, Xinbo Guo, Jianping Pan, Arshad Mehmood Abbasi

**Affiliations:** ^1^Institute of Fruit Tree Research, Guangdong Academy of Agricultural Sciences, Key Laboratory of South Subtropical Fruit Biology and Genetics Resource Utilization, Ministry of Agriculture, Key Laboratory of Tropical and Subtropical Fruit Tree Research, Guangzhou 510640, China; ^2^School of Food Science and Engineering, South China University of Technology, Guangzhou 510641, China; ^3^Department of Environmental Sciences, COMSATS University Islamabad, Abbottabad Campus, 22060, Pakistan

## Abstract

*Clausena lansium* (Lour.) Skeels, commonly known as “wampee,” is an excellent food ingredient of medicinal value. Effects of leaf developmental stages on the composition of phenolics, flavonoids, and antioxidant activity were investigated. Phenolics composition was studied using HPLC-PAD, whereas antioxidant activity was estimated by oxygen radical absorbance capacity (ORAC) and cellular antioxidant activity (CAA) assays. Increase in bound flavonoids, quercetin, and cellular antioxidant activity was observed in bound and free fractions at different stages of leaf development. Predominantly, quercetin and ferulic acid contents were high in free and bound fractions of old leaves. In addition, phenolic components depicted highly significant positive association (*p* < 0.05) with antioxidant activity. Overall, old leaves of wampee have utility value similar to leaf buds, so they could be a more sustainable and economical source of bioactive compounds for commercial application in nutraceutical and pharmaceutical industries.

## 1. Introduction

*Clausena lansium* (Lour.) Skeels commonly known as wampee is an evergreen tree, which is endemic to China. It is commonly cultivated in different parts of southern China including Fujian, Guangdong, Guangxi, and Hainan. Wampee is occasionally grown in India, Sri Lanka, Australia, and the United States [1]. The fruit of wampee has been introduced to southeastern Asia such as Cambodia, Indonesia, Singapore, and Thailand. Wampee fruit is either eaten fresh or served with meat dishes and in preserves, whereas its leaves are used as traditional medicines and food ingredients for cough, asthma, viral hepatitis, dermatological, and gastrointestinal diseases [2]. It has been reported that wampee leaves are rich in functional compounds such as essential oils [3], carbazole alkaloids [4], coumarins [5], triterpenoids [6], and amides, especially clausenamide [7, 8].

More recently, the neuroprotective potential of wampee extract for enhancing memory and to treat Alzheimer's and Parkinson's diseases has been reported [9–12]. The (-) clausenamide extracted from wampee was studied against A*β*-induced neurotoxicity and suggested as a therapeutic agent for Alzheimer's disease [9]. Seven phytochemical compounds including five flavonoids were isolated and identified in wampee leaves [12], including Bu-7 flavonoid, which is a potential compound for treatment of Parkinson's disease and protects PC12 cells against rotenone injury [10].

It is well known that many phytochemicals such as phenolic acids, flavonoids, anthocyanidins, and tannins possess remarkable health-benefiting properties, i.e., antioxidant and anticancer [13, 14], antiviral, and anti-inflammatory activities, and ability to inhibit human platelet aggregation [15–17]. Previous studies were mainly focused on bioactive constituents and their antioxidant activities in wampee stem and fruit [10, 18, 19]. However, phytochemical compounds and antioxidant activity in wampee leaf have rarely been reported so far. Moreover, no systematic study has been conducted on variation in phytochemical contents occurring in wampee leaves during development. Consequently, present study was intended to investigate the change in phytochemical compounds and antioxidant activities in the wampee leaves during different developmental stages of leaf.

## 2. Materials and Methods

### 2.1. Sampling

*Clausena lansium* (Lour.) Skeels leaves of four developmental stages, namely, (i) leaf buds, (ii) young leaves, (iii) mature leaves, and (iv) old leaves, were collected from three 13-year-old trees grown in wampee resources nursery of Institute of Fruit Tree Research, Guangdong Academy of Agricultural Sciences in Guangzhou, China. The samples were stored at −20°C until extraction.

### 2.2. Extraction

Phytochemical contents were extracted by following the method as reported earlier [20, 21]. To extract free phytochemicals, 2 g of each sample was homogenized with 30 mL of 80% chilled acetone (V/V) for 5 mins. Homogenate was centrifuged at 3000 ×g for 5 mins and the supernatant was collected. The residue was extracted again for three times. Supernatants were pooled and evaporated using rotary vacuum evaporator at 45°C till less than 10% of the initial volume. This mixture was then defatted through extracting with hexane (1:2, w/v, 6 times). The free form was reconstituted to 10 mL in Milli-Q water and stored at −40°C before analysis.

To extract bound phytochemicals, the residue from hydrophilic extraction was digested with 10 mL 4 M NaOH at room temperature for 1 hour with shaking. The mixture was acidified to pH 2 using concentrated hydrochloric acid followed by extraction with ethyl acetate. This process was repeated 6 times. The bound form was obtained after evaporating ethyl acetate form and reconstituted in 10 mL Milli-Q water. All extractions were performed in triplicate and extracts were stored at −40°C before analysis.

### 2.3. Determination of Total Phenolics Content

Folin-Ciocalteu method was used to determine the total phenolics content as explained previously [21]. Gallic acid was used as standard. Results were calculated by comparing with standard curve of gallic acid concentrations and expressed as milligram of gallic acid equivalent (GAE) per 100 grams (mg GAE/100 g). Data were reported as mean ± SD of three replicates.

### 2.4. Estimation of Total Flavonoids Content

The flavonoids content of wampee leaves was estimated by the sodium borohydride/chloranil colorimetric method [22], using catechin as standard. Flavonoids content was expressed as milligram of catechin equivalent (CE) per 100 grams (mg CE/100 g). Data were reported as mean ± SD of three replicates.

### 2.5. Phytochemical Composition Analysis by HPLC-PAD

Phenolics and flavonoids components were determined on a Waters RP-HPLC system (Waters Corp, Milford, MA) as reported before [23]. Quercetin, catechin, ferulic acid, chlorogenic acid, and vanillic acid were used as standards. Sample separation was employed with a gradient elution program at the flow rate of 1.0 mL/min and column temperature at 30°C in a Waters Sun Fire™ C18 column (250 mm × 4.6 mm, 5 *μ*m). Each phenolic composition detected was expressed as milligram per 100 grams according to standard curve of each substrate. Results were expressed as mean ± SD (n = 3).

### 2.6. Determination of Total Antioxidant Activity by ORAC Assay

Total antioxidant activity of samples was determined using the oxygen radical absorbance capacity (ORAC) assay as reported previously [24]. Trolox were used as control. The results were expressed as micromole of Trolox equivalent (TE) per 100 grams (*μ*mol TE/100 g). Data were expressed as mean ± SD (n = 3).

### 2.7. Cellular Antioxidant Activity (CAA) Assay

The CAA of wampee leaf extracts was measured as described previously [25]. Human liver cancer cells (HepG2) were used as cell model for analysis. Quercetin was used as standard for calculation. Cellular antioxidant activities of wampee extracts were run with phosphate buffer saline (PBS) washing and no PBS washing. CAA value was expressed as micromole of quercetin equivalent (QE) per 100 grams (*μ*mol QE/100 g). The data was expressed as mean ± SD of three replications.

### 2.8. Statistical Analysis

Statistical analysis was performed using SigmaPlot software 12.3 (Systat Software, Inc., Chicago, IL). Significance of relationships was calculated by multivariate method. Data were analyzed among groups using one-way analysis of variance (ANOVA) and Duncan's multiple comparison posttest using SPSS software 18.0 (SPSS Inc., Chicago, IL). Correlation analyses were performed on the matrix of the 15 quantitative parameters measured on wampee extracts with Pearson Correlation method using SigmaPlot software. P value less than 0.05 was considered statistically significant. All data were reported as mean ± SD of triplicate analyses.

## 3. Results and Discussion

### 3.1. Total Phenolics Content at Different Developmental Stages

**Figure 1:**
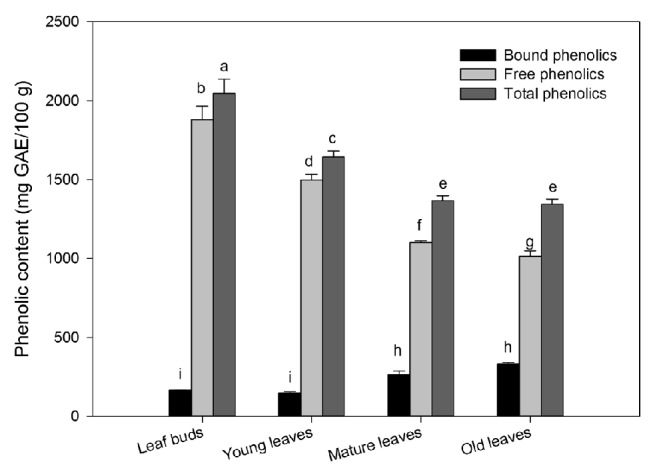
Phenolics content in four developmental stages of wampee leaves (mean ± SD, n = 3). Bars with no letters in common are significantly different (*p*< 0.05).

The results showing total, free, and bound phenolics contents in the leaves of wampee tree collected at four different development stages were mentioned in Figure 1. Leaf buds exhibited the highest total and free phenolics contents (2046 ± 91 and 1880 ± 87 mg GAE/100 g, respectively). However, lowest values were calculated in the mature and old leaves with values of 1367 ± 32 and 1345 ± 32 mg GAE/100 g, respectively (at 34% decline as compared with the initial). In general, there were significant differences (*p*<0.05) in total and free phenolic contents among the four different developing stages. The free phenolic content in the old leaves was the lowest (1013 ± 36 mg GAE/100 g FW) with 46% decline as compared to the initial one. In contrast, the bound phenolic content was significantly increased from 166.0 ± 3.2 to 331.7 ± 10.5 mg GAE/100 g, almost 2-fold increase with leaf developing. These findings revealed significant decline in free and total phenolics but increase in bound phenolics with leaf developing.

The ratio of bound to free phenolics was significantly changed from 8.8 to 32.7% with leaf developing. The free phenolics constituted around 91.9 to 75.3% of the total phenolics which was 9 times higher than the contribution of bound to total phenolics with leaf developing. The average bound and free and total phenolic forms during leaf developing were 227.2, 1373, and 1600 mg GAE/100 g, respectively. It was noted that the bound phenolics were only a small fraction of the total phenolics. Previous studies were mainly focused on the bioactive compounds in fruit and peel of wampee. Prasad et al. [19] reported 330.0 ± 9.9 *μ*g/6 DW, phenolic content, in the peel of wampee using ethyl acetate solvent. Huang reported total phenolic contents in peel, pulp, and seed of wampee fruit (8.40 ± 0.98, 1.30 ± 0.05, and 0.20 ± 0.01 g GAE/100g DW, respectively) [18]. Similarly, 116.1 ± 7.5 mg GAE/100 g of total phenolic content was reported in wampee fruit [10]. Our results indicated that wampee leaves were rich in phenolics, compared to fruits. Therefore, wampee leaves could be useful as insecticides, antimicrobials, and antioxidants and in other industrial applications because of high phenolic content.

### 3.2. Change in Total Flavonoids Content at Different Stages

**Figure 2:**
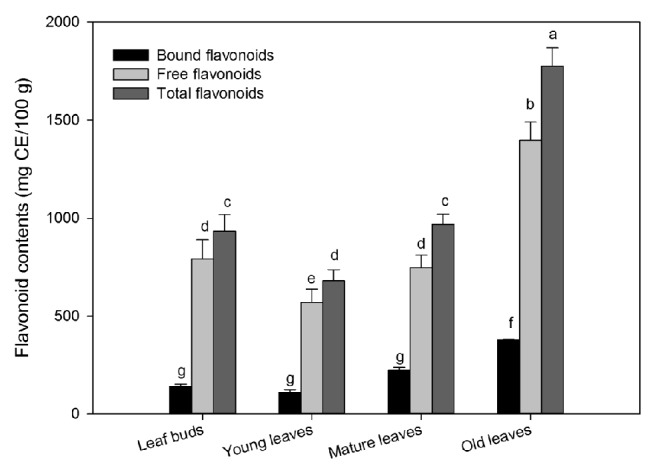
Flavonoids content in four developmental stages of wampee leaves (mean ± SD, n = 3). Bars with no letters in common are significantly different (*p*< 0.05).

The free, bound, and total flavonoids contents in wampee leaves at different stages are given in Figure 2. The old leaf contained highest free flavonoids content 1397 ± 93 mg CE/100 g, followed by leaf buds and mature and young leaves (792.2 ± 96.3, 745.6 ± 64.1, and 570.1 ± 66.6 mg CE/100 g, respectively). The ratio of free flavonoids to total flavonoids content was 84.9, 83.9, 78.6, and 77.1% in leaf buds and young, old, and mature leaves, respectively. The descending order of bound flavonoids form was old leaves > mature leaves > young leaves > leaf buds. The bound flavonoids in mature leaves showed maximum ratio (29.8%) to total flavonoid, followed by old leaves, young leaves, and leaf buds (27.2, 19.2, and 17.8%, respectively).

Following leaf developing, no significant change in the free and bound flavonoids was noted in leaf buds, young leaves, and mature leaves. However, the total flavonoid content was dramatically increased in old leaves (1776 ± 92 mg CE/100 g), with 1.9-fold increase as compared to the initial and 2.6-fold increase as compared with the young leaves. Bound and free forms played important roles for the total flavonoids, which had 3.5- and 2.5-fold increase as compared with the young leaves, respectively. Therefore, leaf developing could promote flavonoids accumulation in old leaves and the fluctuation of flavonoids was contrary to phenolics during leaf developing in wampee.

The ratio of bound to free flavonoids was slightly varied with leaf developing. The contributions of free to total flavonoids was 2~4 times higher than that of bound flavonoids. The average flavonoid contents were 212.9, 876.1, and 1089 mg GAE/100 g as bound, free, and total forms during leaf developing. The results showed that free flavonoids were the main form of the total flavonoids in wampee leaves. An assessment by the number of moles revealed that bound flavonoids contributed 54.5% to bound phenolics, free flavonoids contributed 37.4% to free phenolics, and total flavonoids contributed 41.32% to total phenolics. Consequently, our results suggested that flavonoids were the main composition for phenolics and played important role for phytochemical accumulation in wampee leaves. Previous studies were mainly focused on the phenolic compounds in wampee; flavonoids analysis in wampee have not been reported in the literatures. The flavonoid contents in wampee leaves were reported for the first time in this study.

### 3.3. Phenolics and Flavonoids Composition at Developing Stages of Leaf

Data on phenolics and flavonoids components of wampee leaves at four different developing stages are presented in Table 1. As a whole, two flavonoids (quercetin and catechin) and three phenolic acids (ferulic acid, chlorogenic acid, and vanillic acid) were identified. Among flavonoids, quercetin was almost constant in free and bound forms from leaf buds to mature leaves as 1.45 ± 0.02 mg/100 g (in free form) and 1.42 ± 0.01 mg/100 g (in bound form). In the old yellow leaves 1.70 ± 0.04 and 2.85 ± 0.74 mg/100 g in quercetin content were observed in free and bound forms, 1.2- and 1.9-fold increase as compared with the initial contents, respectively. Catechin was kept consistent in the free form of wampee leaves at four development stages (around 1.9 mg/100 g), whereas it was only detected in the bound form of old leaves with 1.77 ± 0.01 mg /100 g. The variation trends of flavonoid composition were in correspondence with total flavonoid contents in wampee leaves with leaf developing. The results showed that quercetin plays the main role for flavonoids accumulation in wampee during leaf developing.

Among phenolics compounds, ferulic acid, chlorogenic acid, and vanillic acid were detected in free fractions of wampee leaf extract during four development stages, while only ferulic acid was detected in bound form in the young leaves. Ferulic acid was 1.31 ± 0.01 mg/100 g in free fraction of leaf buds; however it was below detection limit in bound fraction. With leaf developing, ferulic acid was increased from 1.75 ± 0.01 to 1.82 ± 0.19 mg/100 g in free and bound fractions of old leaves, respectively. The chlorogenic and vanillic acid were consistent in free form of wampee with leaf developing (average 1.29 and 1.56 mg/100 g, respectively); however they were not detected in bound form. No significant change was observed in the phenolic acids of free fractions at different development stages except ferulic acid, which depicted slight increase in bound form. The variation trends of phenolic acids were in correspondence with phenolics content during leaf developing. Our results indicate that ferulic acid might have a significant role in phenolics accumulation in bound form during different stages of leaf developing.

### 3.4. Variation in Total Antioxidant Activity

**Figure 3:**
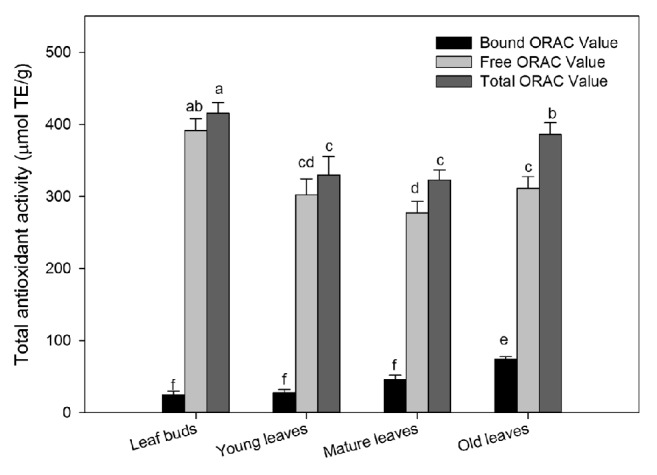
Total antioxidant activity (ORAC) in four developmental stages of wampee leaves (mean ± SD, n = 3). Bars with no letters in common are significantly different (*p*< 0.05).

Results of* in vitro* total antioxidant activity of wampee leaves at four developmental stages as determined by ORAC assay are shown in Figure 3. The ORAC values were highest for total and free forms of leaf buds, followed by old, young, and mature leaves. However, old leaves exhibited maximum ORAC capacity in bounds form, followed by mature leaves, young leaves, and leaf buds. The total ORAC values of wampee leaves ranged from 415.8 ± 14.1 to 323.1 ± 14.1 *μ*mol TE/g in four development stages. However, ORAC value depicted 22% decline from leaf buds to mature leaves and 1.2-fold increase in old leaves compared to mature leaves. The ORAC values in free form of wampee leaves have the same variation trends with the total ORAC values and ranged from 391.2 ± 16.5 to 277.4 ± 16.1 *μ*mol TE/g in four development stages. The free ORAC value dramatically declined 30% from leaf buds to mature leaves and then slightly increased in old leaves (311.5 ± 16.1 *μ*mol TE/g). The ORAC values in bound form of wampee leaves progressively increased from 24.68 ± 5.58 to 74.34 ± 3.44 *μ*mol TE/g during leaf developing. Old leaves have the highest ORAC value in bound form, 3-fold increase as compared with the initial. Therefore, even though the antioxidant activities in free form were declined with leaf developing, the total antioxidant activities of old leaves had no significant changes with the initial one due to increase of antioxidant activities in bound form of wampee leaves.

The ratio of bound to free antioxidant activity increased constantly 6.3 < 9.1 < 16.5 < 23.9 % in leaf buds, young leaves, mature leaves, and old leaves, respectively. However, conflicting trend was noted in the contribution of free to total antioxidant, where leaf buds depicted the highest value, followed by young leaves, mature leaves, and old leaves (94.1, 91.7, 85.9, and 80.7%, respectively). The variation trends of* in vitro *antioxidant activities were in correspondence with phenolics during leaf developing. The results indicated that phenolics were the main sources for* in vitro* antioxidant activities in wampee during the stages of leaf developing. Present results revealed that leaf buds and old leaves have high antioxidant activity. This might be due to high phenolic and flavonoid contents in these parts as estimated in the present investigation. The results suggested that old wampee leaves would have the same utility value with leaf buds, and old leaves would be sustainable and economical resources for food industrial application.

### 3.5. Cellular Antioxidant Activity (CAA)

**Figure 4:**
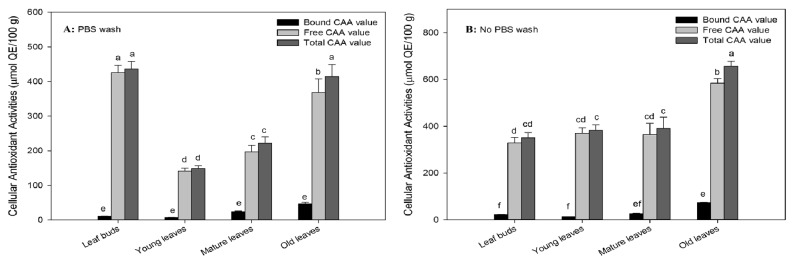
Cellular antioxidant activity (CAA) in four developmental stages of wampee leaves (mean ± SD, n = 3). Bars with no letters in common are significantly different (p< 0.05). A: PBS wash; B: no PBS wash.

The cellular antioxidant activity towards HepG2 cell line was determined in PBS wash and without PBS samples of free, bounds, and total forms of wampee leaves as shown in Figure 4. For PBS wash test, leaf buds had the highest CAA value in free form (425.3 ± 21.8 *μ*mol QE/100 g), 38.6 times higher than the CAA value in bound form of wampee leaves. The free CAA value contributed 97.5% to the total cellular antioxidant activity in leaf buds. The free CAA value was obviously declined to the lowest value in young leaves (142.0 ± 8.0 *μ*mol QE/100 g), 65% decline as compared with the initial. The bound CAA value was also declined to 6.94 ± 0.36 *μ*mol QE/100 g in young leaves, 37% decline as compared with the initial. Meanwhile the contributions of free to total CAA values had no significant changes during the developing stages because of lower percentage of bound form in wampee leaves. The CAA values were dramatically increased from young to old leaves, up to 367.8 ± 39.6 *μ*mol QE/100 g (free CAA value) and 46.47 ± 5.02 *μ*mol QE/100 g (bound CAA value) in old leaves, 2.6 and 6.7 times higher than the cellular antioxidant activities in young leaves, respectively. Meanwhile the ratio of bound to free CAA value was also increased from 2.5% to 12.6% in old leaves, a 5-fold increase as compared with the initial of wampee leaves. The total cellular antioxidant activities had no significant changes between leaf buds and old leaves, even though the values were obviously declined in young and mature leaves with leaf developing. The variation trends of cellular antioxidant activities in PBS wash test were in correspondence with phenolics and* in vitro* total antioxidant activities during wampee leaf developing.

For no PBS wash test, the cellular antioxidant activities of old leaves had the highest CAA values in total, free, and bound forms (657.1 ± 20.4, 583.9 ± 19.3, and 73.09 ± 1.12 *μ*mol QE/100 g, respectively), 1.9, 1.8, and 3.2 times higher than the initial one in wampee leaves. Leaf buds had the lowest CAA value (328.77 ± 22.45 and 350.6 ± 22.1 *μ*mol QE/100 g) in free and total forms, and the CAA values had no significant changes from the developing stages of leaf buds to mature leaves. For the bound form, young leaves had the lowest CAA value (12.84 ± 0.23 *μ*mol QE/100 g), and there were no significant changes from leaf buds to mature leaves during wampee leaf developing too. The contributions of free to bound CAA values were kept consistent in no PBS wash test during wampee leaf developing. The total cellular antioxidant activities were obviously increased in old leaves. The variation trends of cellular antioxidant activities in no PBS wash test were in correspondence with flavonoids during wampee leaf developing.

For the cellular antioxidant activity assay, PBS wash test reflects the fact that the bioactive compounds exhibit antioxidant activities in intracellular status, while no PBS wash test can detect the extracellular and intracellular antioxidant activities of the bioactive compounds [25]. Some flavonoid compounds with glycosides (quercetin-3-glucoside) would be hard to pass through cell membrane into intracellular and play the role in antioxidant activity on membrane to maintain the integrity of cellular structure. Meanwhile the other compounds (quercetin, galangin, and kaempferol) would easily pass through cell membrane and reflect antioxidant activities in intracellular status [26]. Some compounds might transfer to other configurations and exert higher antioxidant activities in intracellular status; for example, galangin has 66.3 ± 1.1 *μ*mol QE/100 *μ*mol in no PBS wash test and 107.8 ± 6.4 *μ*mol QE/100*μ*mol in PBS wash test [26]. In order to evaluate cellular antioxidant activities to a reasonable extent, the ratio of cellular uptake was calculated for bioactive compounds. In our study, leaf buds showed the highest ratio of cellular uptake in free form (129.4%) and the lowest ratio in bound form (50.6%). The cellular uptake ratio of free faction was dramatically declined to the lowest value in young leaves (38.4%), and then a little recovery exists in mature and old leaves (54.2 and 63.0%, respectively). However, the cellular uptake ratio of bound form raised obviously from leaf buds to mature leaves and reached to the highest ratio in mature leaves (93.3%); then the ratio had a little decline in old leaves (63.6%). The present results indicated that mature and old wampee leaves would be excellent sources to extract bioactive compounds as raw material or auxiliary material of food, functional food, or medicines.

### 3.6. Correlations

Associations of phenolic compounds and antioxidant activity in free and bound fractions were studied at different development stages of wampee leaf as shown in Table 2. Free phenolics depicted strong positive correlation with total phenolics and free ORAC value and negative correlation to bound phenolics and bound ORAC value. On the other hand, bound phenolics showed positive correlation with flavonoids, bound ORAC value, and bound cellular antioxidant activities. Free flavonoids were highly correlated to bound flavonoids, total flavonoids, bound ORAC value, and bound cellular antioxidant activity, while having low correlation with free cellular antioxidant activity. Bound flavonoids were also highly correlated to total flavonoids, bound ORAC value, and bound cellular antioxidant activity. Free ORAC value was highly correlated to total ORAC value and free cellular antioxidant activity. Bound ORAC value was highly correlated to bound cellular antioxidant activity, while low correlation was found with free cellular antioxidant activity. No PBS wash cellular antioxidant activity was positively correlated to PBS wash cellular antioxidant activity. The results indicated that phenolics played the main roles for* in vitro* total antioxidant activities, and flavonoids played the main roles for cellular antioxidant activities in wampee during leaf developing.

## 4. Conclusion

Wampee leaves were found as an excellent source of bioactive compounds with high antioxidant activities. A spectacular increase in bound phenolics, flavonoids, and cellular antioxidant activities was observed with the development of leaf at different stages. This study suggests that old leaves of wampee have same utility value like leaf buds and could be used as sustainable and economical source of bioactive compounds for nutraceutical and pharmaceutical industrial applications.

## Figures and Tables

**Table 1:** Phytochemical composition in wampee leaves at different developing stages.

Compounds	Conjugated ways	Leaf buds	Young leaves	Mature leaves	Old leaves
Quercetin	free	1.46 ± 0.01	1.34 ± 0.03	1.45 ± 0.02	1.70 ± 0.04*∗*
	bound	1.52 ± 0.01	1.49 ± 0.01	1.42 ± 0.01	2.85 ± 0.74*∗*
Catechin	free	1.93 ± 0.06	1.87 ± 0.04	1.91 ± 0.08	1.92 ± 0.02
	bound	ND	ND	ND	1.77 ± 0.01
Ferulic acid	free	1.31 ± 0.01	1.49 ± 0.01	1.66 ± 0.03	1.75 ± 0.01*∗*
	bound	ND	1.29 ± 0.03	1.28 ± 0.02	1.82 ± 0.19*∗*
Chlorogenic acid	free	1.27 ± 0.01	1.27 ± 0.01	1.39 ± 0.07*∗*	1.26 ± 0.01
	bound	ND	ND	ND	ND
Vanillic acid	free	1.52 ± 0.01	1.55 ± 0.01	1.66 ± 0.01	1.51 ± 0.01
	bound	ND	ND	ND	ND

Unit: mg/100 g. ND means not detected; *∗* indicates significant difference at *p*< 0.05.

**Table 2:** Correlation of phenolics, flavonoids, and antioxidant activities in wampee leaves (Pearson test, *p* < 0.05).

Variables	FP	BP	TP	FF	BF	TF	F-ORAC	B-ORAC	T-ORAC	F-N CAA	B-N CAA	T-N CAA	F-CAA	B-CAA	T-CAA
FP	1	−0.84	**0.99**	−0.54	−0.76	−0.60	0.79	−0.83	0.45	0.45	−0.64	−0.66	0.28	−0.77	0.17
BP		1	−0.75	0.84	**0.95**	0.88	−0.37	**0.92**	0.04	0.04	0.88	0.83	0.23	**0.96**	0.34
TP			1	−0.43	−0.67	−0.49	0.86	−0.76	0.56	0.56	−0.54	−0.59	0.40	−0.68	0.30
FF				1	0.91	**0.99**	0.01	0.86	0.43	0.43	**0.97**	**0.92**	0.58	0.89	0.66
BF					1	**0.95**	−0.27	**0.97**	0.18	0.18	**0.97**	**0.91**	0.36	**0.98**	0.46
TF						1	−0.06	**0.90**	0.37	0.37	**0.99**	**0.93**	0.53	**0.93**	0.62
F-ORAC							1	−0.39	0.89	0.89	−0.11	−0.21	0.71	−0.27	0.64
B-ORAC								1	0.06	0.06	**0.93**	**0.92**	0.20	**0.96**	0.31
T-ORAC									1	1	0.32	0.21	0.87	0.17	0.85
F-N CAA										1	0.32	0.21	0.87	0.17	0.85
B-N CAA											1	**0.95**	0.47	**0.95**	0.57
T-N CAA												1	0.29	0.89	0.39
F-CAA													1	0.31	**0.99**
B-CAA														1	0.42
T-CAA															1

FP: free phenolics, BP: bound phenolics, TP: total phenolics, FF: free flavonoids, BF: bound flavonoids, TF: total flavonoids, F-ORAC: free ORAC value, B-ORAC: bound ORAC value, T-ORAC: total ORAC value, F-N CAA: free no PBS wash CAA value, B-N CAA: bound no PBS wash CAA value, T-N CAA: total no PBS wash CAA value, F-CAA: free PBS wash CAA value, B-CAA: bound PBS wash CAA value, and T-CAA: total PBS wash CAA value.

## Data Availability

All data are included in the article.
